# Surgical management of atrioesophageal fistula after catheter ablation of atrial fibrillation: A French nationwide study

**DOI:** 10.1016/j.xjon.2024.09.010

**Published:** 2024-09-21

**Authors:** Ludovic Dupautet, Guillaume Lebreton, Gabriel Saiydoun, Thierry Bourguignon, Sébastien Frey, Christophe Beaufreton, Géraud Galvaing, Sébastien Cambier, Marc Filaire, Laura Filaire

**Affiliations:** a Department of Thoracic and Endocrine Surgery, Centre Jean Perrin, Clermont-Ferrand, France; b Department of Cardiothoracic Surgical, Hospital La Pitié-Salpêtrière, Paris, France; c Division of Cardiothoracic Surgery and Transplantation, Hospital Henri Mondor, Paris, France; d Department of Cardiac Surgery, Tours University Hospital, Tours, France; e Department of Digestive Surgery and Liver Transplantation, Nice University Hospital, Nice, France; f Department of Cardiovascular and Thoracic Surgery, University Hospital, Angers, France; g Department of Biostatistic, University Hospital Gabriel Montpied, Clermont-Ferrand, France

**Keywords:** atrial fibrillation, atrioesophageal fistula, esophageal perforation, esophagectomy, pericardo-esophageal fistula

## Abstract

**Objective:**

The study objective was to assess the efficacity of different surgical strategies for atrioesophageal fistula after catheter ablation of atrial fibrillation.

**Methods:**

Between January 2010 and April 2023, all patients with a diagnosis of atrioesophageal fistula or pericardo-esophageal fistula after catheter ablation of atrial fibrillation were analyzed retrospectively from the French database EPITHOR. Patients without surgical management were excluded.

**Results:**

Eighteen patients were included, 15 with atrioesophageal fistula and 3 with pericardo-esophageal fistula. Median follow-up was 89.5 days with an overall survival of 50%. Five patients underwent esophageal stenting, 2 as a bridge-to-esophagectomy with 50% of survival and 3 in association with esophagus and left atrial direct repair with 66% survival. Primary esophageal repair with flap coverage was performed in 8 patients with 25% survival, most of them with sepsis and neurological failure. Seven patients had an esophagectomy with 71% survival, only 2 of them having a neurological failure. Among them, 5 patients underwent a restorative surgery and are still alive. Four patients had a retrosternal colon interposition, and 1 patient had an esogastric anastomosis. Risk factors for death were neurological failure (hazard ratio [HR], 4.91, 95% CI, 0.95-25.22; *P* = .0057) in univariate analysis and sepsis (HR, 6.25, 95% CI, 1.17-33.3; *P* = .032) in multivariate analysis. Esophagectomy tended to offer a survival benefit (HR, 0.163, 95% CI, 0.019-1.340; *P* = .092). The use of cardiopulmonary bypass did not significantly impact survival (HR, 1.953, 95% CI, 0.392-9.719; *P* = .413).

**Conclusions:**

Aggressive surgical strategies for managing atrioesophageal fistula are mandatory to offer the best chance of survival.

## Graphical Abstract

**Figure:**
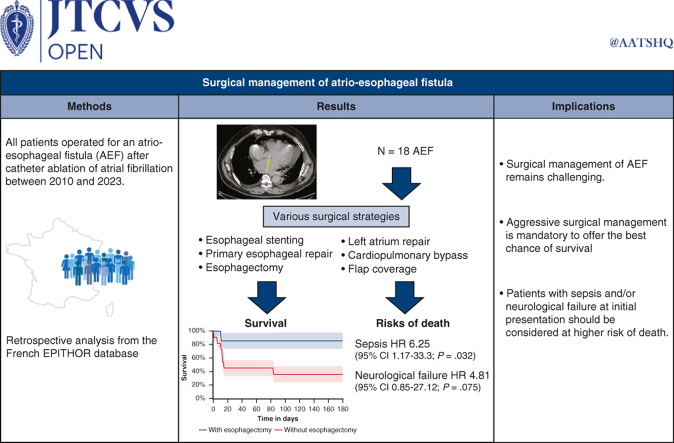

**Figure:**
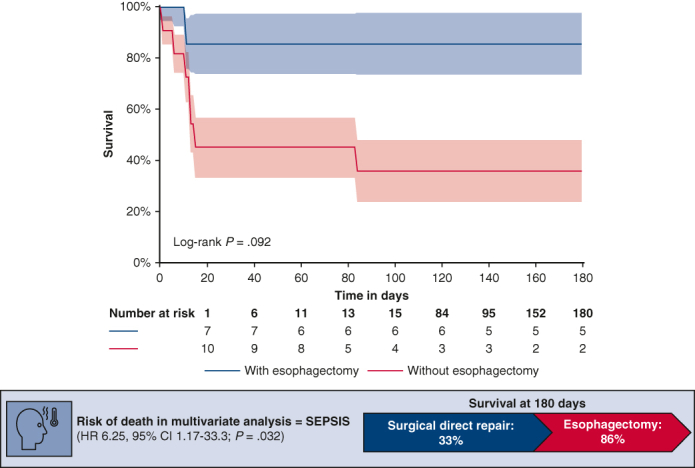
Survival at 180 days did not differ between esophagectomy and PER.

**Central Message**

Aggressive surgical management in AEF is mandatory to offer the best chance of survival.

**Perspective**

Every patient readmitted to the hospital for any symptoms within the 4 weeks after CA of AF should be considered to have an AEF until proven otherwise. Novel energy sources such as pulse-field ablation are emerging, potentially making AEF even more rare.

Atrioesophageal fistula (AEF) is the most feared and life-threatening complication after catheter ablation (CA) of atrial fibrillation (AF). Its incidence remains stable at approximately 0.02% to 0.04% despite technical improvements and novel energy sources over time.^1^, ^2^, ^3^ Overall mortality of AEF has been reported from 55% to 65%.^2^^,^^4^ Among the various therapeutic options (medical treatment, endoscopic treatment alone or associated with surgery, and upfront surgical treatment), surgery offers a clear benefit in survival compared with endoscopic or medical treatment alone.^1^^,^^2^^,^^5^, ^6^, ^7^ Although many studies on AEF have assessed the prognostic of patients between medical treatment or surgery,^3^^,^^5^^,^^6^^,^^8^ few of them give details on the surgical strategy. Conservative or radical surgical options are reported for the esophagus perforation management by review of the literature compiling cases or monocentric studies,^7^^,^^9^, ^10^, ^11^ without consensus. However, to our knowledge, there is no national experience on AEF management focusing on surgical technique.

Thus, we aimed to present our surgical experience and results on the management of AEF after CA of AF in a French cohort.

## Patients and Methods

The present study is in accordance with the Declaration of Helsinki and was approved by the Institutional Review Board of the French Society of Thoracic and Cardiovascular Surgery on November 10, 2022 (CERC-SFCTCV-2023-04-25_28215). A written informed consent for publication was sent to each patient or their relatives.

### Population and Study Design

A retrospective observational cohort study was conducted from January 2010 to April 2023. Inclusion criteria were patients aged more than 18 years with a diagnosis of AEF or pericardo-esophageal fistula (PEF) after CA of AF who were treated with a surgical approach. Because patients’ data were extracted from the French surgical database EPITHOR, patients treated medically or with endoscopic strategies alone were excluded. Patients were identified by sending an email to the 68 French cardiothoracic surgical units, all participating in the EPITHOR database.^12^ EPITHOR database was created in 2003, available via a secured website. Collected data of the patients of each center are anonymous and used for the evaluation and improvement of surgical practices as well as clinical research.^12^ Twenty-one centers replied (30.8%), and among them, only 6 tertiary cardiothoracic surgical units had eligible patients (8.8%).

### Data Collection

Baseline characteristics, comorbidities, AF treatment-related variables, AEF subtypes, clinical and paraclinical exams, surgical strategies (surgical approach, use of cardiopulmonary bypass [CBP], management of the esophageal perforation, flap interposition), and follow-up data were collected by each participating center.

The primary outcome was the survival after AEF treated surgically. The secondary outcome was the comparison of the different surgical strategies to highlight risk factors for mortality.

### Statistical Analysis

All statistical analysis was performed using STATA software version 15 (StataCorp LLC). Given the exploratory nature of the analysis, no adjustment for type I error was made. A bilateral type I error risk of 5% was considered in all analyses. Categorical variables are described by frequencies and associated percentages, and quantitative variables are presented as mean and SD or median and interquartile range, depending on their statistical distribution. Normality was assessed through graphical analysis. A survival analysis was conducted on the time to death. A Cox-type model was used to assess the main dependencies of survival time. The center effect was also studied.

## Results

Eighteen patients met eligibility criteria. The mean age was 58 ± 13 years. Sixteen patients were male (88.9%). Fifteen patients had a diagnosis of AEF (83.3%), and 3 patients had a diagnosis of PEF (16.7%). Baseline characteristics are shown in Table 1. No difference was observed between centers.

**Table 1:** Clinical description of atrioesophageal fistula cases (n = 18)

Comorbidities	N (%) or mean ± SD
Left ventricular ejection fraction, %	53.6 ± 9.1
Comorbidities	
Hypertension	9 (50)
Smoke	5 (27.8)
Diabetes	3 (16.7)
Gastroesophageal reflux	1 (5.6)
Obesity	4 (22.2)
AF subtypes	
Paroxysmal	3 (16.7)
Persistent	8 (44.4)
Permanent	7 (38.9)
Type of CA	
Radiofrequency	17 (94.4)
Cryoablation	1 (5.6)

Categorical variables are presented as numbers (percentages). Quantitative variables are presented as mean (±SD). *AF*, Atrial fibrillation; *CA*, catheter ablation.

**Figure 1:**
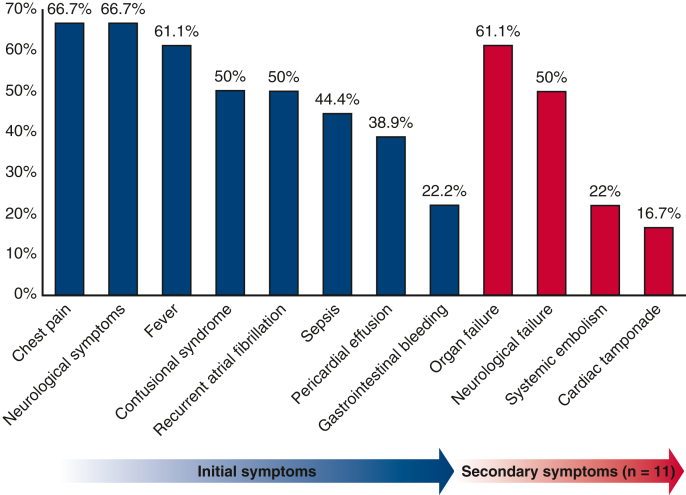
Clinical presentation. Neurological symptoms were stroke, transient ischemic attack, meningitis, and seizures. Neurological failure and systemic embolism were defined as coma and embolism in any territory except encephalic, respectively.

Figure 1 represents the incidence of initial and secondary symptoms. All AEF diagnoses were made preoperatively. Table 2 details the paraclinical exams. Diagnosis was made for 10 patients (55.5%) based on the chest computed tomography (CT). Reported abnormalities were free air between the left atrium and the esophagus, or free air within the pericardium associated with pericardial effusion. Esophageal instrumentations (transesophageal echocardiogram or esophagogastroduodenoscopy [EGD]) were performed in half of the patients and allowed diagnosis in 6 patients (33%). No clinical degradation was reported after any endoscopic procedure. Retrospectively, reinterpretation of the first chest CT could have set the diagnosis of AEF in 7 patients (38.9%). Among them, 2 patients (11.1%) were diagnosed by EGD.

**Table 2:** Paraclinical exams

Paraclinical exams	n	Sensibility
Chest CT with contrast	17 (94)	65
Chest CT with contrast esophagogram	3 (16.7)	66.6
Cerebral imaging	12 (67)	17
Transthoracic echocardiography	16 (89)	6
Transesophageal echocardiography	1 (5.6)	0
EGD	8 (44)	75

Values are presented as numbers (percentages) or percentages. *CT*, Computed tomography; *EGD*, esophagogastroduodenoscopy.

**Figure 2:**
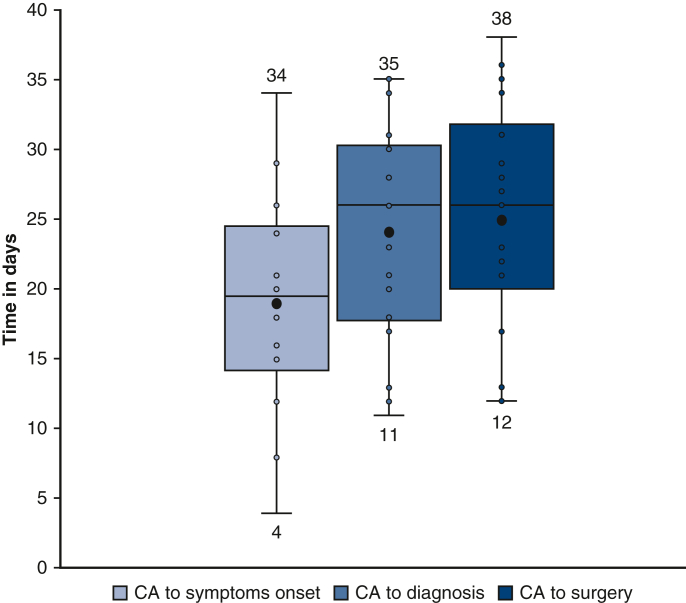
Temporal trends from CA to surgery. Boxplots represents the various temporal trends from CA to surgery. The *lower* and *upper borders* of the *box* represent the lower and upper quartiles. The *middle horizontal line* represents the median. The lower and upper whiskers represent the minimum and maximum values. *Black circle* represents the mean time. Each time value for the 18 patients has been plotted and represented by *circle*. *CA,* Catheter ablation.

Figure 2 illustrates the different median times from CA to symptoms onset, diagnosis, and surgery. Median time from symptoms to diagnosis was 3 days (2-8.75 days). Surgical treatment was realized within the 24 hours after the diagnosis in more than 75% of patients (median time, 0.50 days (0-1 days)). Diagnosis was first made in 44.4% of the patients (n = 8). Patients were first admitted to a reference center in 66.7% of cases (n = 12).

### Surgical Outcomes

**Figure 3:**
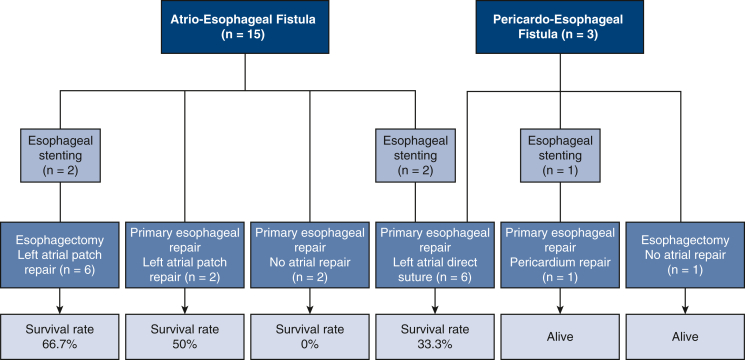
Tree diagram of treatments. This diagram illustrates the different surgical strategies used for patients categorized by the technique used to manage esophageal and atrial (or pericardial) perforations. No surgical strategy was statistically associated with an increased survival.

Surgical strategies for each patient are detailed in Table 3 and summarized in a tree diagram (Figure 3). Five patients (27.7%) underwent esophageal stenting. For 2 of them (11.1%) (patients 4 and 13), AEF was diagnosed by EGD. Esophageal stenting was performed as a bridge-to-esophagectomy in 2 patients (11.1%) (patients 13 and 15) due to persistent leakage and mediastinitis, both requiring a redo surgery for postoperative complications. One patient died, and 1 is still alive. In patients 4, 5, and 18, esophageal stenting was associated with esophageal and left atrium direct repair and flap interposition. Two patients underwent reoperation and are still alive, whereas patient 5 died 15 days after surgery. Seven patients (38.9%) underwent esophagectomy, among whom a restorative surgery was performed in 5 patients (71.4%). Four patients underwent a retrosternal colon interposition. Patient 7 underwent a partial esophagectomy (around the fistula) without esophagostomy and left atrial exploration due to a PEF (Video 1). An early restorative surgery at postoperative day 10 was performed by esogastric anastomosis. Six patients (85.7%) underwent reoperation for complications (n = 2) or a restorative surgery (n = 5). Two patients died (28.6%). Eight patients (44.4%) underwent a surgical treatment with primary esophageal repair (PER) and flap interposition, without previous esophageal stenting. Six of them (75%) also had a left atrium repair by direct suture or patch repair. Only 2 patients (25%) underwent reoperation for complications. Six patients (75%) died postoperatively. Flap interposition was systematically realized in the absence of esophagectomy. CPB was used in 12 patients (66.6%) whatever surgical option was considered for esophageal repair.

**Table 3:** Atrioesophageal treatment (n = 18)

N	Age, y	Sex	AEF subtypes	Presenting symptoms	Imaging for diagnosis	Esophageal stenting	Surgical strategies	Complications	Restorative surgery	Vital status	Cause of death
1∗	34	M	AEF	Sepsis AF recurrence PeriE	Chest CT	-	Sternotomy, CPB, LA patch repair, thoracotomy, PER, flap interposition	Redo surgery	-	Deceased	MOF
2	72	M	PEF	ThP PeriE	EGD	-	Sternotomy, CPB, LA direct suture, thoracotomy, PER, flap interposition	Redo surgery	-	Deceased	NR
3∗	65	M	AEF	Sepsis AF recurrence	Brain CT	-	Sternotomy, LA direct suture, PER, flap interposition	NR	-	Deceased	NR
4	58	M	PEF	ThP Sepsis PeriE	Chest CT	Yes	Sternotomy, CPB, pericardium direct suture, thoracotomy, PER, flap interposition	Redo surgery	-	Alive†	-
5∗	65	M	AEF	ND - Seizure Fever ThP	Chest CT contrast esophagogram	Yes	Sternotomy, CPB, LA direct suture, thoracotomy, PER, flap interposition	NR	-	Deceased	Coma
6∗	64	M	AEF	ND Fever - ThP	TTE	-	Thoracotomy, PER, flap interposition	NR	-	Deceased	Coma
7	69	F	PEF	AF recurrence PeriE ThP	Chest CT	-	Thoracotomy, partial esophagectomy, flap interposition	NR	Yes‡	Alive§	-
8∗	49	M	AEF	ND - Sepsis AF recurrence Splenic embolism	Brain CT	-	Thoracotomy, CPB, LA direct suture, PER, flap interposition	NR	-	Deceased	CPA
9	55	M	AEF	ND Sepsis AF recurrence	EGD	-	Clamshell, CPB, LA direct suture, PER, flap interposition	NR	-	Alive†	-
10∗	66	M	AEF	ND Sepsis ThP	Chest CT	-	Clamshell, CPB, LA direct suture, PER, flap interposition	NR	-	Deceased	Coma
11∗	70	F	AEF	ND AF recurrence	EGD	-	Clamshell, CPB, LA patch repair, PER, flap interposition	NR	-	Alive†	-
12	44	M	AEF	ND Fever - ThP AF recurrence Systemic embolism	Chest CT	-	Thoracotomy, CPB, LA patch repair, esophagectomy	NR	Yes‖	Alive†	-
13∗	65	M	AEF	ND AF recurrence	EGD	Yes	Thoracotomy, LA patch repair, esophagectomy	Redo surgery	-	Deceased	MOF
14∗	52	M	AEF	ND ThP	Chest CT	-	Thoracotomy, LA patch repair, esophagectomy	NR	Yes‖	Alive†	-
15	31	M	AEF	Fever - ThP AF recurrence	Chest CT	Yes	Thoracotomy, CPB, LA patch repair, esophagectomy	Redo surgery	Yes‖	Alive†	-
16	64	M	AEF	Transient ischemic attack Seizure Fever	Chest CT	-	Thoracotomy, CPB, LA patch repair, esophagectomy	NR	Yes‖	Alive†	-
17	42	M	AEF	ND Sepsis PeriE - ThP	Chest CT	-	Thoracotomy, CPB, LA patch repair, esophagectomy	NR	-	Deceased	Cerebral hemorrhage
18	80	M	AEF	Epigastralgia	Chest CT with contrast esophagogram	Yes	Thoracotomy, PER, flap interposition	Redo surgery	-	Alive§	-

*AEF*, Atrioesophageal fistula; *AF*, atrial fibrillation; *CT*, computed tomography; *CPB*, cardiopulmonary bypass; *LA*, left atrium; *PER*, primary esophageal repair; *MOF*, multiorgan failure; *PEF*, pericardo-esophageal fistula; *ThP*, thoracic pain; *PeriE*, pericardial effusion; *EGD*, esophagogastroduodenoscopy; *NR*, not reported; *ND*, neurological defect; *TTE,* transthoracic echocardiogram.

∗ Patient with neurological failure (coma) at initial presentation.

† With sequela.

‡ Restorative surgery with esogastric anastomosis.

§ Without sequela.

‖ Restorative surgery with retrosternal colon interposition.

Among the 9 patients with neurological failure at initial presentation, only 2 patients (patients 13 and 14) underwent an esophagectomy (28.5%) (Table 3), whereas the other patients underwent a PER (63.6%).

The mean lengths of stay in the intensive care unit and hospitalization were 27.9 ± 24.6 days and 53.9 ± 46.1 days, respectively. A center effect was observed concerning the different surgical approaches and esophageal perforation management: thoracotomy (*P* = .002), sternotomy (*P* = .008), clamshell (*P* = .0029), and esophagectomy (*P* = .002). No center effect was observed concerning the use of CPB (*P* = .471) and AEF subtypes (*P* = .082). Time to diagnosis was not influenced by a center effect or a suspected diagnosis at admission.

### Survival

**Figure 4:**
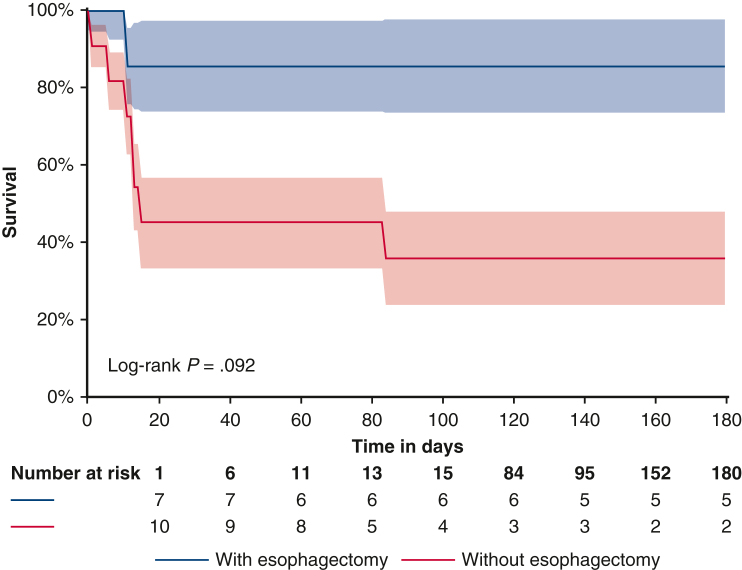
Survival curve according to the use of esophagectomy. Survival at 180 days after esophagectomy is 86% versus 33% in case of other surgical treatments (*P* = .092). 95% CI.

The mean follow-up time was 768.2 days with a median of 89.5 days. Overall approximate survival was 50% (n = 9). The Kaplan–Meier survival was 60% at 15 days and 55% at 84 days, which was the time of last fatal event. No differences in survival were seen among the different surgical approaches. Overall survival appeared to be better, despite no significant difference, in case of PEF than AEF (hazard ratio [HR], 1.9; 95% CI, 0.23-15.81; *P* = .54), the use of an esophageal stent (HR, 0.258; 95% CI, 0.03-2.1; *P* = .2), and esophagectomy (HR, 0.163, 95% CI, 0.019-1.340; *P* = .092) with 86% survival at 180 days (Figure 4). The use of CPB did not impact survival (HR, 1.953, 95% CI, 0.392-9.719; *P* = .413).

In univariate analysis for risk factors for death, only sepsis (HR, 6.398, 95% CI, 1.265-32.359; *P* = .025) and neurological failure (HR, 4.905, 95% CI, 0.954-25.218; *P* = .0057) were statistically relevant. In multivariate analysis, a significant independent risk factor for death was sepsis (HR, 6.25, 95% CI, 1.17-33.3; *P* = .032), whereas neurological failure tended to negatively impact the prognosis with a 4-time increase in the risk of death (HR, 4.81, 95% CI, 0.85-27.12; *P* = .075).

## Discussion

**Figure 5:**
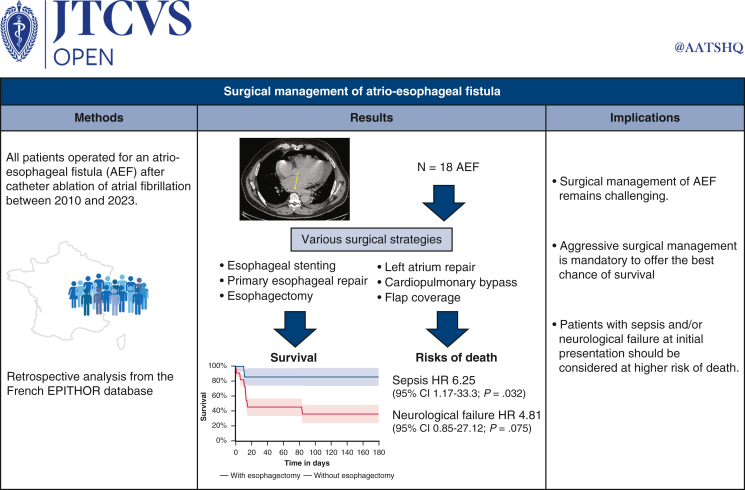
Graphical abstract depicting the study design.

This French nationwide study aimed to evaluate the different surgical strategies available for the management of AEF after CA of AF from a surgical database. To the best of our knowledge, this is the largest national cohort on this topic currently available in the literature. Our main findings highlight the wide range of surgical management approaches available that vary from one center to another, confirming the need for aggressive surgical management for esophageal perforation, the absence of worse mortality in the use of CPB, and an increased risk of death associated with sepsis and neurological failure at initial presentation (Figure 5).

Our cohort of 18 patients appears to be a real-life representation of medical reality according to a previous French study that lacked surgical data.^1^ Our results confirm that AEF is an iatrogenic, ischemic, noncontained, and late thoracic esophageal perforation occurring in the 4 weeks after CA of AF and managed lately with a mean time from symptoms onset to diagnosis of 5 days. The presence of fever, chest pain, and neurological symptoms should be considered as an AEF until proven otherwise after CA of AF. Moreover, neurological symptoms and failure are known to be associated with a latter AEF onset compared with other patients^8^ and a higher risk of mortality,^4^ as in our multivariate analysis.

Chest CT sensitivity was inferior to other studies,^1^^,^^2^^,^^7^^,^^8^ although the retrospective reinterpretation increased the sensitivity. Some hypotheses might explain these results: First, patients were treated in various centers all around France with first patients’ admission in secondary hospitals. Second, the warning symptoms of AEF are not specific and can mimic other pathologies such as meningitis, stroke, or pericarditis. This second point is of major concern because clinical presentation directs toward diagnostic hypotheses on which depend the paraclinical exams. Third, the under-recognition of the main diagnostic hypothesis (AEF) by emergency physicians can underline their lack of knowledge of AEF after CA of AF or a lack of their formation by interventional cardiologists. Moreover, a high proportion of esophageal instrumentation was performed as a diagnostic tool and realized before any discussion with a surgical team. EGD is not recommended in case of suspected AEF^13^ because it can increase systemic air emboli through the fistula and aggravates clinical symptoms in 28% of patients.^4^ Repeated chest CT and a better knowledge of AEF by emergency physicians/radiologists could increase the sensitivity, reducing the time to diagnosis and ensuring an urgent management.

### Surgical Strategy

The survival benefit of a surgical management of AEF is well established^1^^,^^2^^,^^5^, ^6^, ^7^ as the surgical treatment of esophageal perforation according to “patient-related–factors.”^13^ However, literature on the efficacy of the different surgical strategies is sparse.^9^, ^10^, ^11^^,^^14^^,^^15^ We found that surgical approaches differed from patient to patient and center to center but had no consequences on mortality.^7^

CPB was used in two-thirds of patients, without a difference in cannulation strategies between centers and did not significantly impact survival, contrary to Amirkhosravi and colleagues^7^ who found that CPB was associated with better survival (84.8% vs 15.2%; *P* < .001). In a report of 5 AEF cases, the only survivor without neurological sequela after surgical treatment of AEF was the patient with an internal left atrial repair using CPB.^11^ Benefits of CPB in AEF rely on preventing air or septic emboli during surgical repair and manipulation, crossclamping,^7^ and resection of necrotic left atrium tissue to avoid secondary septic emboli or endocarditis whatever the cannulation strategies.^11^ In case of PEF or doubtful AEF, exploration of the left atrial wall might be preferable due to the potential worse course of patients with unrecognized left atrial perforation in balanced with the benefit of CPB in AEF.^7^^,^^11^

Esophageal stenting was used in 5 patients as a bridge-to-esophagectomy or in association with PER, without increasing the risk of death. In the POTTER-AF study, patients receiving an interventional treatment (stenting or surgery) had a lower mortality rate and less stroke during clinical course.^2^ However, worse survival was reported after esophageal stenting alone.^1^^,^^4^^,^^5^^,^^7^^,^^8^ No difference in survival was found between endoscopic treatment in association with surgery in comparison with surgery alone. Thus, esophageal stenting should be associated with surgical management in a “bridge-to-surgery” strategy.

PER reinforced by flap without previous stenting was performed in 8 patients with a high mortality rate (75%) contrary to the result of a recent review of literature where PER tended to offer better results than esophagectomy, although statistically nonsignificant.^7^ This high mortality rate after PER might be explained by the following hypotheses. Our patients operated on PER seems to be more severe than those undergoing esophagectomy. In fact, 28% and 63% of our patients with esophagectomy and PER, respectively (Table 3), presented an initial neurological failure, known to be a poor prognostic factor,^4^ whereas only 12% of their patients presented an initial neurological failure, without information according to the surgery. Moreover, they reported a 2-stage surgery (first stage performed in emergency by sternotomy with left atrium repair and PER; second stage performed the day after by right thoracotomy to interpose a muscle flap^7^), whereas our reported cases, treated by PER, were managed in the same way but in a different surgical timing: 1-stage surgery by a one (right thoracotomy) or double surgical approach (sternotomy and right thoracotomy).

Esophagectomy was performed in 7 patients (38.9%), most of them still alive (71%). This nonstatistically significant survival benefit in favor of esophagectomy might be overestimated and explained by patients with less severe disease at initial presentation contrary to PER. However, most of our patients received restorative surgery within 6 months essentially by colon interposition in a retrosternal route and are still alive. The retrosternal route seems to be better than the posterior mediastinum, which can be hostile after AEF. The number of redo surgeries for complications were similar in the patients who underwent esophagectomy and PER. In view of our results and surgical guidelines^13^^,^^16^ on esophageal perforation management, esophagectomy might be a valuable radical damage control surgery as an alternative to PER in AEF management, especially when surgical management is realized late.

Partial esophagectomy involving the AEF area might be a good alternative to esophagectomy. It allows a restorative surgery by esogastric anastomosis keeping the colonic transplant in case of failure and avoiding the retrosternal route after a previous sternotomy. This technique was realized in only 1 of our patients. No other case is found in the literature.

Bipolar esophageal exclusion was not performed in our study but could allow effective control of AEF in a damage control surgery while maintaining the possibility of further restorative surgery, although esophageal mucocele can occur on the esophageal remnant.^17^

### Survival

Overall survival was 50%, which is in line with the literature^2^^,^^7^ with a higher mortality rate in the first 90 postoperative days. In our series, only esophagectomy tended to positively influence the survival. Risk factors of mortality were sepsis and coma. Other risk factors have been reported: stenting or medical management alone,^2^^,^^8^ the absence of CBP,^7^ gastrointestinal bleeding, and age more than 60 years.^5^

### Strength and Limitations

This study has some limitations. The nonresponse of approximately two-thirds of cardiothoracic centers to the inclusion process introduced a selection bias. Only 18 patients were included, which can be explained by the rare incidence of AEF but appears to be real life compared with the previous French survey.^1^ Many surgical strategies were assessed with just a few patients in each group, decreasing statistical power and limiting statistical analysis. Moreover, our results on esophagectomy should be interpreted with caution because patients seemed to have less severe disease at initial presentation than those with PER. Comparison of surgical results on quality of life was not possible because no data were available for patients who were still alive.

## Conclusions

Aggressive surgical strategies for managing AEF are needed to offer the best chance of survival for patients.

## Conflict of Interest Statement

The authors reported no conflicts of interest.

The *Journal* policy requires editors and reviewers to disclose conflicts of interest and to decline handling or reviewing manuscripts for which they may have a conflict of interest. The editors and reviewers of this article have no conflicts of interest.

## Supplementary Data

Video 1 The case of patient 7. Video available at: https://www.jtcvs.org/article/S2666-2736(24)00258-4/fulltext.
