# Supplementary figures and images for: Surgical management of atrioesophageal fistula after catheter ablation of atrial fibrillation: A French nationwide study
Source: JTCVS Open. 2024 Sep 21;22:476–84. doi: 10.1016/j.xjon.2024.09.010 (PMC11704549; doi:10.1016/j.xjon.2024.09.010)

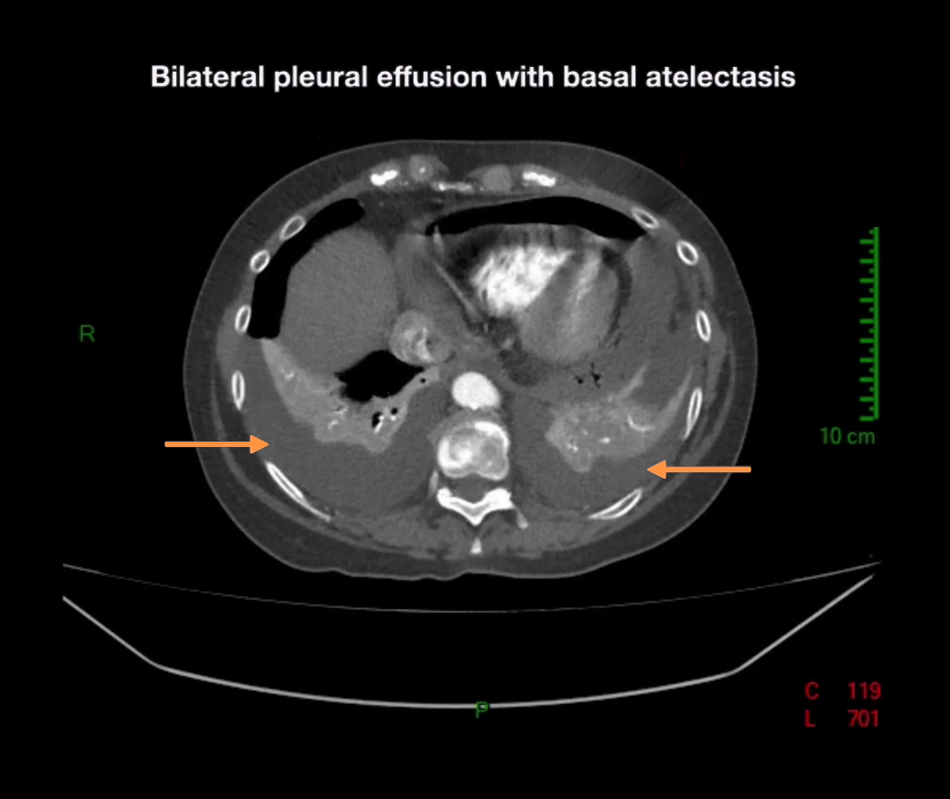

Supplement: Video 1 — The case of patient 7. Video available at: https://www.jtcvs.org/article/S2666-2736(24)00258-4/fulltext. [file fx2.jpg]
